# Supplementary material for: X-ray phase contrast reveals soft tissue and shell growth lines in mollusks
Source: Commun Biol. 2024 Jan 3;7:17. doi: 10.1038/s42003-023-05457-y (PMC10764734; doi:10.1038/s42003-023-05457-y)
Supplement: Supplementary file 2 — Supplementary Information [file 42003_2023_5457_MOESM2_ESM.pdf]

Supplementary information: X-ray phase contrast reveals soft tissue and shell growth lines in mollusks

Ilian Häggmark[1,*], Masato Hoshino[2], Kentaro Uesugi[2], and Takenori Sasaki[1]

[1]The University Museum, The University of Tokyo, 7-3-1 Hongo, Bunkyo-ku, Tokyo, 113-0033, Japan
[2]Japan Synchrotron Radiation Research Institute (JASRI/SPring-8), 1-1-1 Kouto, Sayo, Hyogo, 679-5198, Japan
[*]ilianh@um.u-tokyo.ac.jp

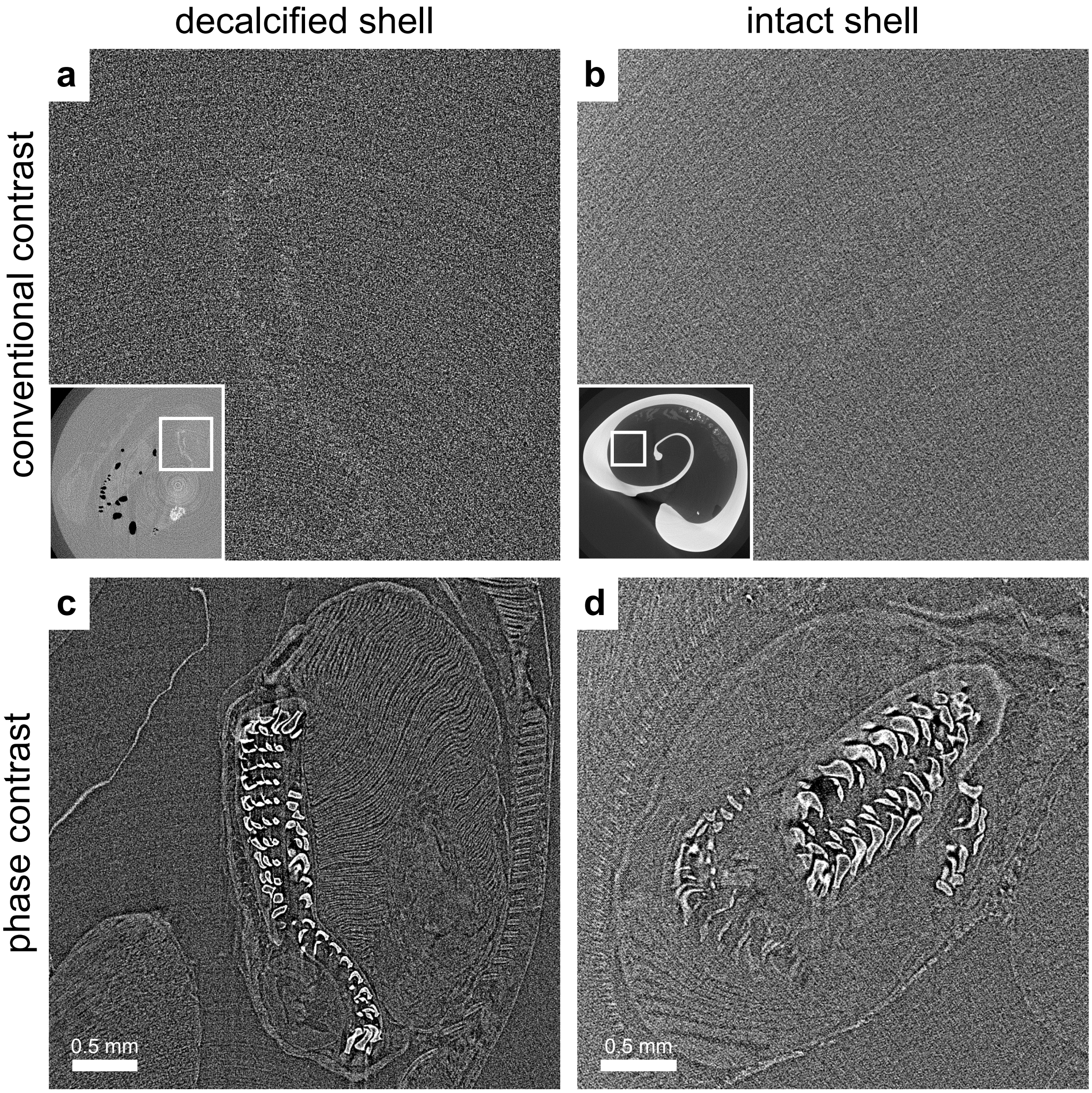

Supplementary Figure 1. Difference in contrast for the four settings used. The radula of the *Monetaria annulus* are shown with and without decalcification and with and without phase contrast. This figure shows the significant difference between attenuation-based contrast imaging and phase contrast imaging under various conditions (such as the state of the shell). It should be noted that decalcification enables further processing such as dehydration which may substantially increase the contrast in the images shown in the left column. Furthermore, the X-ray energy is optimized for the images in the right column. A direct comparison between the two columns can thus be misleading.
